# Moderate heart rate-matched hypoxic exercise: autonomic and cardiovascular responses to different degrees of hypoxic stress

**DOI:** 10.1007/s00421-025-05910-2

**Published:** 2025-07-26

**Authors:** Alessandro Fornasiero, Alicia González Represas, Andrea Zignoli, Federico Stella, Mark Rakobowchuk, Laurent Mourot

**Affiliations:** 1 CeRiSM, Sport Mountain and Health Research Centre, University of Verona, Rovereto, Italy; 2 Department of Neurosciences, Biomedicine and Movement Sciences, University of Verona, Verona, Italy; 3 Universidade de Vigo. Grupo de Investigación Fisioterapia, Departamento de Biología Funcional y Ciencias de La Salud, Facultad de Fisioterapia Campus A Xunqueira S/N, Universidade de Vigo, 36005 Pontevedra, Spain; 4 Department of Industrial Engineering, University of Trento, Trento, Italy; 5 Department of Biological Sciences, Faculty of Science, Thompson Rivers University, Kamloops, Canada; 6 Université de Franche-Comté, SINERGIES, 25000 Besançon, France

**Keywords:** Hypoxia; baroreflex sensitivity, Hypoxic exercise, Cardiac autonomic activity, Autonomic nervous system

## Abstract

**Purpose:**

This study aims to assess the impact of HR-matched exercises under varying hypoxic stress levels on exercise and post-exercise autonomic and cardiovascular responses.

**Methods:**

Twelve moderately aerobically trained healthy men (mean age: 23 ± 2 years, height: 179 ± 8 cm, weight: 71.2 ± 9.9 kg, BMI: 22.2 ± 2.2 kg/m^2^, VO_2_max: 53.1 ± 4.2 mL/min/kg) completed an interval exercise session at 75% of their normoxic maximum heart rate (75%HRmax) under three hypoxic conditions: FiO_2_ = 16.2% (2000 m a.s.l; H16), FiO_2_ = 14.3% (3000 m a.s.l; H14), and FiO_2_ = 12.6% (4000 m a.s.l; H12). Each session included 5 min of seated rest, a 5-min sub-maximal load warm-up, and five 5-min work intervals with 1-min passive recovery periods.

**Results:**

During hypoxic exercise, RMSSD decreased significantly following the first bout coinciding with an increase in heart rate. The RMSSD increase during 60-s recovery intervals was significantly lower after the 4th and 5th bouts compared to the 1st and 2nd bouts (*p* < 0.05). At 15 min post-exercise, mean RR, systolic blood pressure and stroke volume decreased. No changes were observed in cardiac output or baroreflex sensitivity. At 60 min post-exercise, SDNN, RMSSD, mean arterial pressure and diastolic blood pressure increased significantly compared to 15 min post-exercise. No condition or interaction differences were found.

**Conclusion:**

Despite the decreased oxygen saturation with increased hypoxia levels, HR-matched interval exercise induced similar cardiac and autonomic responses across all hypoxic conditions. Baseline cardiac autonomic function and hemodynamics recovered within 60 min with no impact of hypoxia on baroreflex sensitivity.

## Introduction

Hypoxic training involves exercising under conditions of reduced oxygen concentration or partial pressure of oxygen using hypoxia masks, altitude simulation chambers, or hypoxia training devices. Research about exercise performed under hypoxic conditions (HE) has increased exponentially in recent years. Understanding its physiological effects is necessary not only to better understand how hypoxic physical training affects performance, but also to develop possible applications including sports science or rehabilitation.

The autonomic nervous system plays a crucial role in maintaining the cardiovascular stability during and after exercise, and thereby blood flow and oxygen delivery to the tissues (Dufour et al. 2010). However, it is not well known how the autonomic nervous system interacts in this regard in HE. During HE, sympathetic nervous system activity increases as well as vasodilator-mediated vasodilation of blood vessels is triggered (Casey and Joyner 2012; Stickland et al. 2009), but an excessive increase in sympathetic activity may result in endothelial dysfunction, increased peripheral vascular resistance, higher heart rate and overload of ventricular function. (Grassi and Drager 2024).

In addition, baroreflex sensitivity can be significantly reduced during HE, meaning that blood vessels are unable to adapt to changes in blood pressure. As a result, blood pressure is more unstable both during exercise and in post-exercise recovery (Bourdillon et al. 2023). The recovery phase reflects the dynamics of parasympathetic reactivation and sympathetic withdrawal, both essential for cardiovascular recovery. During this phase, and especially under hypoxic conditions, autonomic recovery may exhibit alterations affecting hemodynamic stability and baroreflex sensitivity, which may not be evident during exercise. (Fornaseiro et al. 2018).

Full restoration of autonomic balance is a complex, time-dependent process. Although recovery begins within the first few seconds to minutes after exercise, this initial phase primarily reflects a rapid and transient response, characterized by immediate changes in heart rate and sympathetic activity. In healthy, physically active individuals, the duration of post-exercise autonomic recovery may range from approximately 10–15 min to up to 60 min, depending on the intensity of the exercise performed. The 10–15 min post-exercise time-point represents an early but more stable recovery phase, during which parasympathetic reactivation and sympathetic withdrawal become more evident. By 60 min post-exercise, autonomic balance is typically expected to return to baseline, allowing for the evaluation of whether recovery is complete or if persistent alterations remain (Seiler et al. 2007a, 2007b).

Hypoxic exercise has been shown to reduce both metabolic and mechanical load while modulating autonomic nervous system activity. These effects may help optimize the cardiovascular response to physical exertion, particularly in trained individuals, offering a wide range of training possibilities (Millet et al. 2010). Previous studies suggest that the autonomic nervous system's response is influenced by both the exercise protocol and the level of hypoxia (Fornasiero et al. 2021, 2019), particularly by the intensity of these stimuli. This intensity could play a critical role in modulating the response, as it may limit the benefits of hypoxic training and/or negatively impact cardiovascular function. Besides, hypoxic exercise performed at normoxic work rates (NE) results in a significant increase in sympathetic response and reduced baroreflex sensitivity which contribute to a delayed restoration of autonomic balance and altered cardiovascular responses during post-exercise recovery.

However, moderate hypoxic exercise matched to heart rate (HR) does not seem to impose a greater cardiorespiratory stimulus and may not lead to greater post-exercise autonomic impairment. Current evidence suggests that by controlling HR, HE can induce ventilatory responses comparable to normoxic exercise (NE), with reduced metabolic and mechanical demand. Moreover, recent findings indicate that moderate hypoxic exercise matched to approximately 75% of maximal heart rate (HRmax) and a Fraction of Inspired Oxygen (FiO_2_) of 14.2%, simulating an altitude of approximately 3000 m above sea level (m a.s.l.), can elicit cardiac autonomic responses similar to those observed during NE sessions (Fornasiero et al. 2021, 2019).

The full impact of HR-matched hypoxic exercise conducted at various levels of hypoxic stress, particularly at simulated altitudes exceeding 3000 m, on exercise and post-exercise autonomic and cardiovascular responses has not been fully investigated. Further research is needed to thoroughly understand the hemodynamic implications, as well as to evaluate the potential risks and benefits associated with exercising under different hypoxic conditions.

The aim of this study is to evaluate the effect of performing moderate HR-matched exercise at different hypoxic levels (% FiO2) on acute autonomic and cardiovascular responses during exercise and at 15–60 min post recovery period. We hypothesized that moderate HR-matched exercise would result in similar autonomic responses during both exercise and the early and late post-exercise phases, regardless of the degree of hypoxic stress.

## Methods

### Participants

Twelve moderately aerobically trained healthy men (mean age: 22.8 ± 2.2 years, height: 179 ± 8 cm, weight: 71.2 ± 9.9 kg, BMI: 22.2 ± 2.2 kg/m2, VO_2_max: 53.1 ± 4.2 mL/min/kg) participated in this study. None of the participants exhibited clinical evidence of cardiovascular, metabolic, or musculoskeletal diseases. Prior to data collection, all participants received detailed information about the experimental protocol and provided written informed consent. They were instructed to abstain from consuming caffeine, alcohol, and engaging in high-intensity exercise for 24 h before each experimental session. The experimental protocol was approved by the local Human Research Ethics Committee (CPP Est I 2016-A00511-50) and was carried out in accordance with the Declaration of Helsinki.

Protocol

Each participant attended the laboratory on four occasions, consisting of one preliminary evaluation session and three experimental sessions, all scheduled at the same time of day within a 4-week period. All tests were conducted under controlled laboratory conditions (21 °C, 65% relative humidity). Different levels of hypoxia, under normobaric conditions, were induced during the experimental sessions by adjusting the fraction of inspired oxygen (FiO2) using a Cloud 9 hypoxic generator (Sporting Edge, Basingstoke, UK). This system prevented us from collecting respiratory and gas exchanges information during exercise. Exercise sessions were performed on a Monark 818E cycle ergometer (Stockholm, Sweden), equipped with a SRM device (SRM, PowerControl, Jülich, Germany), with participants maintaining a constant pedaling cadence of 90 revolutions/min under visual feedback.

During the preliminary session participants anthropometrical characteristics were measured and habituation to the experimental settings was performed by doing a 5 min of NE at a target HR of 120 bpm. In the subsequent three visits, participants completed an interval exercise protocol under three different degrees of hypoxia in a random order: FiO_2_ = 16.2% (≈2000 m a.s.l), FiO_2_ = 14.3% (≈3000 m a.s.l), and FiO_2_ = 12.6% (≈4000 m a.s.l). Each exercise session included 5 min of seated rest, a 5-min sub-maximal constant load warm-up, followed by five 5-min work intervals interspersed with 1-min passive recovery periods (i.e., 5 x (5-min work: 1-min rec)). Participants were exposed to hypoxia only during the exercise interventions. The measurements performed before and after were conducted in a normoxic environment. This protocol was adopted to gather key information about the physiological and cardiac autonomic responses to interval-type hypoxic exercise (Fornasiero et al. 2019).

To ensure comparable relative intensity across conditions, workload was gradually adjusted during the first interval to achieve the target heart rate of 75% of maximum heart rate (HRmax) calculated as 220 minus age, and then maintained at a constant level throughout the exercise session (Garber et al. 2011; MacIntosh et al. 2021). This approach allowed heart rate to vary naturally in response to hypoxia, reflecting the cardiovascular and autonomic load under the different levels of hypoxia.

Throughout exercise and recovery phases, R-R intervals were continuously recorded using a Polar RS800CX HR monitor (Polar, Kempele, Finland), while pulse oxygen saturation (SpO2) was monitored using fingertip pulse oximetry (WristOx2 3150, Nonin Medical, Minneapolis, MN, USA). Participants also reported their individual rating of perceived exertion (RPE) at the end of each exercise bout using the Borg Category Ratio Scale (CR100) (Borg and Borg 2002).

Autonomic nervous system and hemodynamic assessments were conducted immediately before (PRE) exercise and at two different time-points after exercise (POST-15 min, POST-60 min), in a quiet room under normoxic conditions (21 °C, 65% relative humidity). The 15 min post-exercise time-point was selected to assess whether ANS has achieved partial recovery, while the 60 min post-exercise was chosen to evaluate if full recovery of ANS balance has been achieved. Continuous measurements of beat-by-beat blood pressure and R-R intervals were obtained using a Portapres® device (Finapres Medical System, Amsterdam, The Netherlands) and a Polar RS800CX HR monitor (Polar, Kempele, Finland), respectively, while participants were in a supine position for 10 min.

### Data analysis

The R-R intervals were extracted using Polar Precision Performance Software (Polar, Kempele, Finland) and saved as.txt files. Signal artifacts were removed using a moderate error correction filter, ensuring that all R-R interval time series exhibited minimal noise (identified errors < 5%). Post-exercise heart rate recovery (HRR) and heart rate variability (HRV) indices were calculated using a customized script in MATLAB (MathWorks Inc., USA). HRR indices were derived from the absolute differences between HR at the end of exercise (HRbout), calculated as the mean of the last 30 s, and HR values at 30 s (HR30) and 60 s (HR60) into recovery (HRR30 and HRR60, respectively), averaged over 5 s intervals (Bucheit et al. 2007). Additionally, normalized HRR (%HRR) was calculated as the relative decline in HR expressed as a percentage of HRbout (nHRR30 and nHRR60). The time-varying vagal-related index root mean square of successive differences of R-R intervals (RMSSD) was calculated for the last 30 s of exercise (RMSSDbout) and for each of the two 30-s segments of recovery (RMSSD30 and RMSSD60) (Peçanha et al. 2017).

Resting HRV analysis was performed using Kubios HRV software (Version 2.1, Biosignal Analysis and Medical Imaging Group, Kuopio, Finland) (Tarvainen et al. 2014). HRV indices were computed based on data from the final 5 min of the 10-min supine resting period. Time-domain HRV indices included RMSSD, standard deviation of normal-to-normal RR intervals (SDNN), and percentage of successive R-R intervals differing more than 50 ms from the previous interval (pNN50). Frequency-domain HRV indices, including low frequency (LF, 0.04–0.15 Hz), high frequency (HF, 0.15–0.4 Hz), and total power (TP, 0–0.4 Hz), were calculated using Fast Fourier Transform (FFT) (Task Force of the European Society of Cardiology et al. 1996). Additionally, respiratory frequency (Rf) during exercise was estimated from the HF peak using an autoregressive model (Tarvainen et al. 2014).

Inter-beat interval (IBI) and beat-to-beat systolic (SBP) and diastolic (DBP) blood pressure values were obtained using Beatscope Software (TNO-TPD, Biomedical Instrumentation). Mean blood pressure values were calculated from the last 5 min of a 10-min supine period. Post-exercise hypotension (PEH) was defined as the absolute difference between SBP at POST and SBP at PRE (PEH = SBP POST – SBP PRE). Baroreflex sensitivity (BRS) was assessed using beat-by-beat SBP and IBI values. Transfer function analysis (TFA) of gain, phase, and coherence between spontaneous oscillations in SBP and IBI was performed using linearly interpolated and resampled data at 2 Hz (Zhang et al. 2009). Baroreflex sensitivity was also evaluated using the sequence method, which identifies of at least three consecutive beats (sequences) with defined changes in SBP followed by changes in IBI, with a minimum correlation coefficient of 0.85 (Pinna et al. 2015). Positive and negative sequences were averaged separately to compute mean gain values of positive (BRSSeq +) and negative (BRSSeq-) sequences, providing a comprehensive representation of blood pressure control in both directions.

### Statistical analysis

The data are presented as means ± standard deviations (SD). Normal distribution was assessed using the Shapiro–Wilk test. In cases where data did not follow a normal distribution, a natural logarithm transformation (Ln) was applied to achieve normality and enable parametric statistical comparisons. For comparisons of exercise responses, a two-way ANOVA with repeated measures was conducted, with “condition” (FiO_2_ 16.2%, FiO_2_ 14.3%, and FiO_2_ 12.6%) and “time” (number of bouts) as within factors. Pre- and post-exercise responses were compared using a two-way ANOVA with repeated measures, with “condition” (FiO_2_ 16.2%, FiO_2_ 14.3%, and FiO_2_ 12.6%) and “time” (PRE-POST15min-POST60min) as within factors. Post hoc analysis was performed using the Sidak test to determine specific differences between conditions or time points. Statistical analyses were conducted using SPSS software (SPSS Inc, Chicago, Illinois, USA). Repeated measures correlations between cardiac autonomic indices and physiological variables were conducted using the R package labeled “rmcorr” (Bakdash and Marusich 2017). Correlation coefficients were evaluated as follows: > 0.1 small; > 0.3 moderate; > 0.5 large; > 0.7 very large; and > 0.9 extremely large (Hopkins et al. 2009). The level of statistical significance was set at p < 0.05.

## Results

### Exercise sessions

**Fig. 1:**
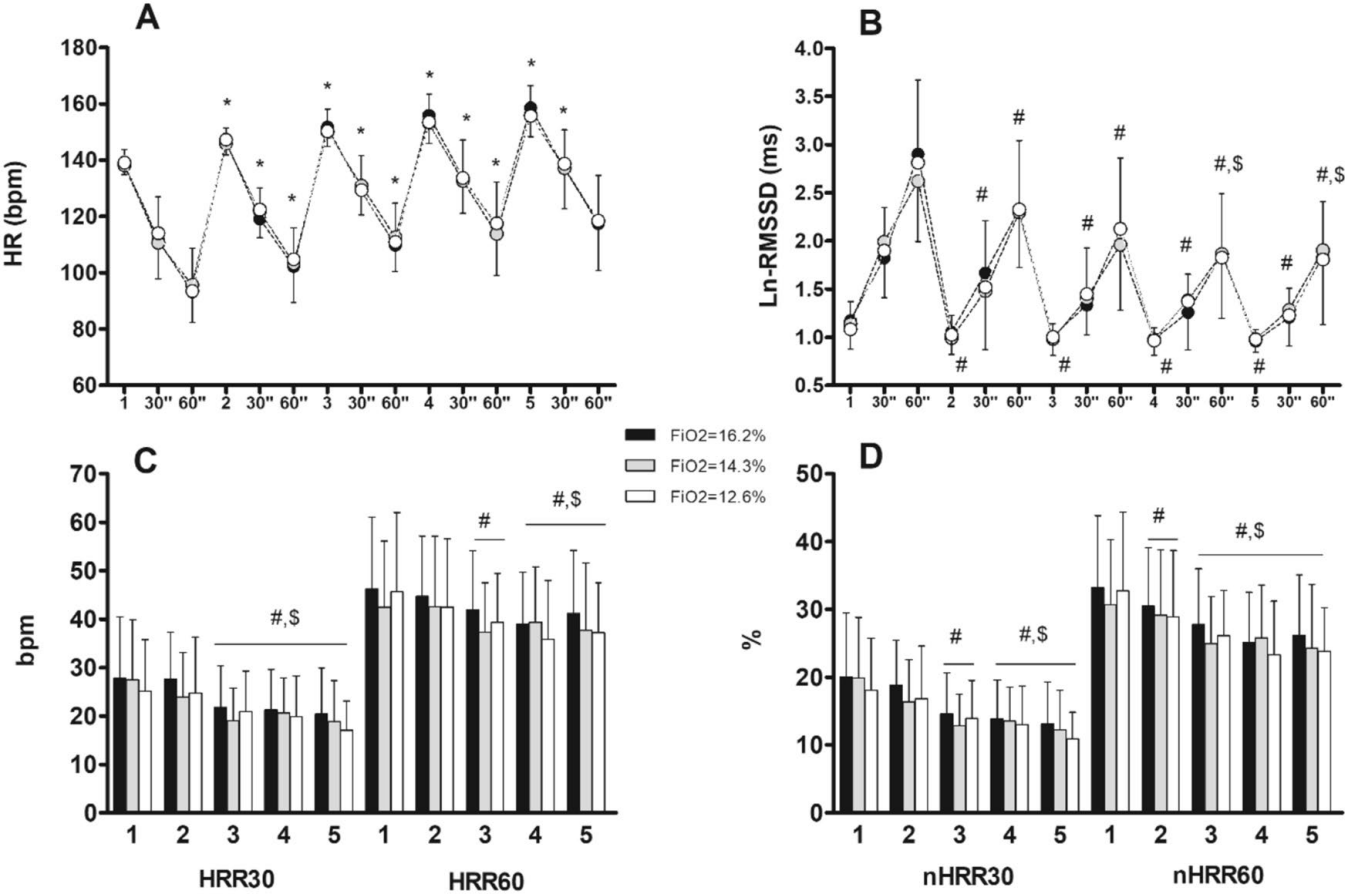
Cardiac autonomic modulation responses during the three interval exercise sessions (5x (5-min exercise bout; 1-min passive recovery)). Black, gray and white circles and bars represent hypoxic exercise with 16.2% (≈2000 m), 14.3% (≈3000 m) and 12.6% (≈4000 m) of fraction of inspired oxygen, respectively. **A** Heart Rate (bpm); **B** Natural-logarithm transformation of the root mean square of successive differences of R–R intervals (Ln-RMSSD); **C** Heart rate recovery (bpm) at 30 and 60 s of recovery; **D** Normalized Heart Rate Recovery (nHHR) at 30 and 60 s of recovery calculated as (%HRR = HRR/HRbout × 100); time effect: *: ≠ from previous bout; #: ≠ from 1st bout; $: ≠ from 2nd bout

During the exercise sessions, the workload was 172 ± 27 W, 156 ± 19 W, and 132 ± 26 W in the FiO_2_ 16.2%, FiO_2_ 14.3%, and FiO_2_ 12.6% conditions, respectively. A significant effect of the “condition” was observed on session workload (*p* = 0.001). Workload was significantly reduced in FiO_2_ 12.6% compared to FiO_2_ 14.3% (−15%, *p* = 0.047) and H16 (−23%, *p *= 0.001), with no significant difference between FiO_2_ 16.2% and FiO_2_ 14.3% (−9%, *p* = 0.119). Cardiac autonomic modulation responses during the three exercise sessions are illustrated in Fig. 1.

A significant effect of time (*p* < 0.001) on exercising HR was observed, independent of condition (p = 0.476) or interaction effects (*p* = 0.495). The exercising heart rate increased significantly with each successive bout (general time effect, *p* < 0.05). Similarly, significant general time effects (*p* < 0.001) were noted for HR30 and HR60, with no significant condition (*p* = 0.844 and *p* = 0.886, respectively) or interaction effects (*p* = 0.669 and *p* = 0.777, respectively). Throughout the exercise session, both HR30 and HR60 showed a consistent increase (each recovery period differed from the previous one, *p* < 0.05), except for HR60 after the 5th bout (*p* > 0.05).

Similarly, a significant effect of time (*p* < 0.001) on RMSSD was observed, with no significant condition (*p* = 0.815) or interaction effects (*p* = 0.341). RMSSD significantly decreased after the 1st bout (general time effect, *p* < 0.05), with similar trends observed for RMSSD30 and RMSSD60 (*p* < 0.001). Post-hoc analysis revealed a decrease in both RMSSD30 and RMSSD60 after the 2nd, 3rd, 4th, and 5th bouts compared to the 1st bout (time effect, all *p* < 0.05), and in RMSSD60 after the 4th and 5th bouts compared to the 2nd bout (time effect, all *p* < 0.05). However, there were no significant condition or interaction effects for RMSSD30 and RMSSD60 (*p* = 0.592, *p* = 0.847, *p* = 0.439, and *p* = 0.532, respectively).

Regarding HRR, only a significant effect of time (*p* < 0.001) was reported for both HRR30 and HRR60, with no significant condition (*p* = 0.240 and *p* = 0.290, respectively) or interaction effects (*p* = 0.904 and *p* = 0.717, respectively). HRR30 significantly decreased after the 3rd, 4th, and 5th bouts compared to the 1st and 2nd bouts (all p < 0.05), while HRR60 decreased after the 3rd compared to the 1st bout, and after the 4th and 5th bouts compared to the 1st and 2nd bouts (all *p* < 0.05).

Similarly, only a significant effect of time (*p* < 0.001) was reported for nHRR30 and nHRR60, with no significant condition (*p* = 0.364 and *p* = 0.374, respectively) or interaction effects (*p* = 0.881 and *p* = 0.995, respectively). nHRR30 significantly decreased after the 3rd bout compared to the 1st bout, and after the 4th and 5th bouts compared to the 1st and 2nd bouts (all *p* < 0.05). nHRR60 significantly decreased after the 2nd bout compared to the 1st bout, and after the 3rd, 4th, and 5th bouts compared to the 1st and 2nd bouts (all *p* < 0.05).

**Fig. 2:**
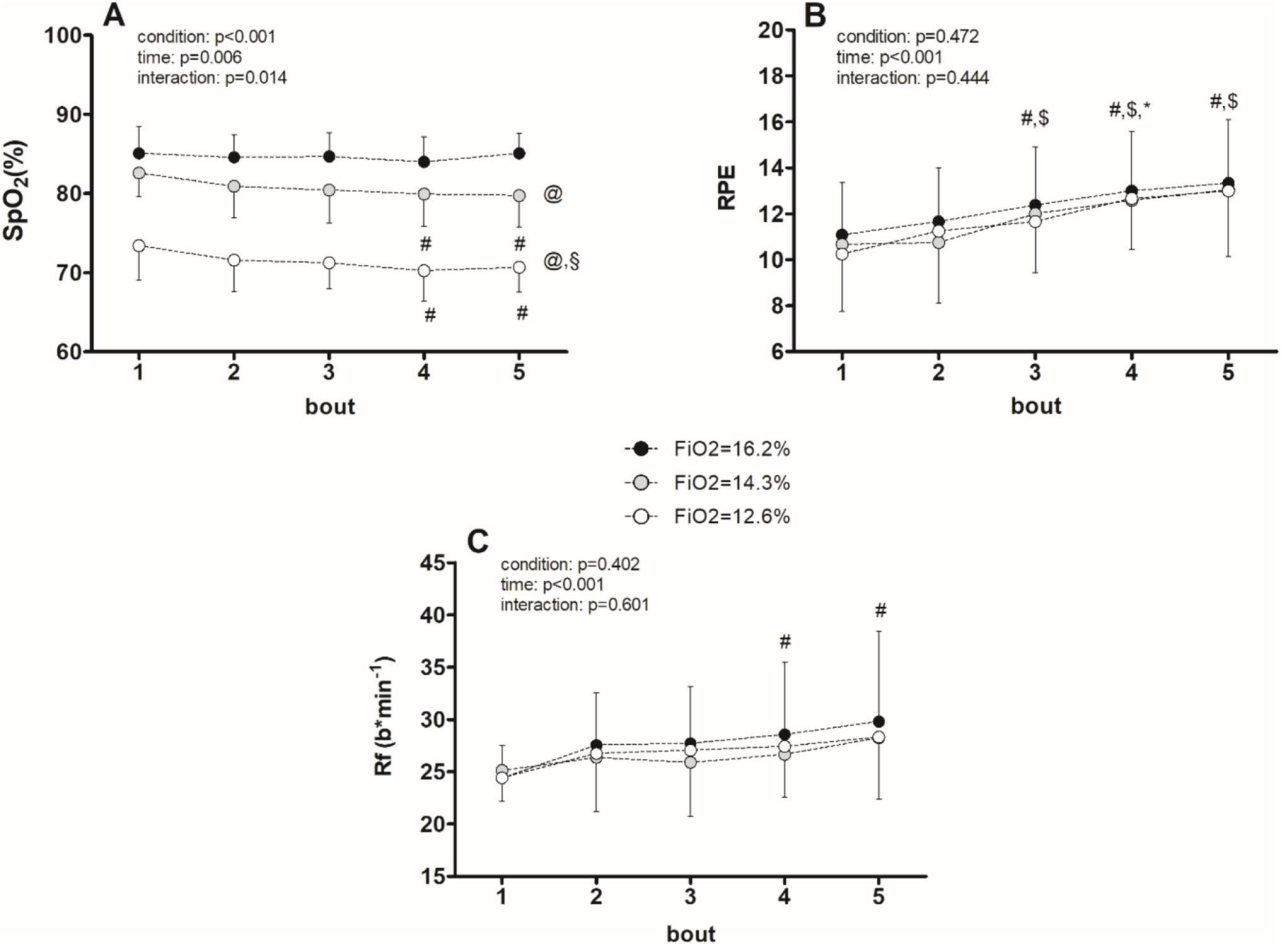
Physiological exercise responses during the three exercise protocols. Black, grey and white circles represent hypoxic exercise with 16.2% (≈2000 m), 14.3% (≈3000 m) and 12.6% (≈4000 m) of fraction of inspired oxygen, respectively. SpO_2_, pulse oxygen saturation; RPE, Rate of Perceived Exertion; Rf, Respiratory frequency; time effect: *: ≠ from previous bout; #: ≠ from 1st bout; $: ≠ from 2nd bout; condition effect: @ ≠ 2000 m; §: ≠ 3000 m

Figure 2 illustrates the physiological and perceptual responses (SpO_2_, respiratory frequency (Rf), and rating of perceived exertion (RPE)) observed during the three exercise sessions. Significant effects were noted for SpO_2_, with time (*p* = 0.006), condition (*p* < 0.001), and time*condition interaction (*p* = 0.014) all showing statistical significance. SpO_2_ significantly decreased with the escalating hypoxic stress levels (condition effect, all *p* < 0.05). Moreover, SpO_2_ significantly declined during the 4th and 5th bouts compared to the 1st bout in FiO_2_ 14.3%, and FiO_2_ 12.6% conditions. In terms of Rf, a significant effect of time (*p* < 0.001) was observed, with no significant condition (*p* = 0.402) or interaction effects (*p* = 0.601). Rf exhibited a notable increase during the 4th and 5th bouts compared to the 1st bout across all hypoxic stimuli.

Similarly, there was a general time effect observed for RPE without significant condition or interaction effects. RPE increased during the 3rd, 4th, and 5th bouts compared to the 1st and 2nd bouts, and after the 4th bout compared to the 3rd bout (all *p* < 0.05) across all hypoxic stimuli.

The complete results of the repeated measures correlations are presented in Table 1. Overall, cardiac autonomic recovery indices obtained during interval-training exercise were found to have negative correlations with exercising HR (*p* < 0.05). Specifically, HRR30 and HRR60 demonstrated negative correlations, with moderate and small strengths, respectively, in relation to HRbout. Notably, the magnitudes of these correlations increased when considering normalized HRR values, showing large and moderate strengths for nHRR30 and nHRR60, respectively.

**Table 1:** Repeated measure correlations between cardiac autonomic recovery indices and exercise physiological responses

		HR				SpO_2_				Rf				RPE			
		*r*	Descriptor	95%CI		*r*	Descriptor	95%CI		*r*	Descriptor	95%CI		*r*	Descriptor	95%CI	
Recovery	HR30	**0.89**	Very large	0.85	0.92	**−0.16**	Small	**−**0.31	**−**0.01	**0.47**	Moderate	0.34	0.58	**0.55**	Large	0.44	0.65
	HR60	**0.81**	Very large	0.75	0.86	**−0.17**	Small	**−**0.31	**−**0.02	**0.40**	Moderate	0.26	0.52	**0.50**	Large	0.38	0.60
	HRR30	**−0.46**	Moderate	**−**0.58	**−**0.33	**0.17**	Small	0.01	0.31	**−0.22**	Small	**−**0.36	**−**0.07	**−0.24**	Small	**−**0.38	**−**0.09
	HRR60	**−0.27**	Small	**−**0.41	**−**0.13	**0.18**	Small	0.03	0.32	**−**0.08	Trivial	**−**0.23	0.07	**−**0.12	Small	**−**0.27	0.03
	nHRR30	**−0.57**	Large	**−**0.67	**−**0.46	**0.18**	Small	0.02	0.32	**−0.28**	Small	**−**0.41	**−**0.13	**−0.31**	Moderate	**−**0.44	**−**0.16
	nHRR60	**−0.48**	Moderate	**−**0.59	**−**0.36	**0.19**	Small	0.04	0.33	**−0.19**	Small	**−**0.34	**−**0.04	**−0.26**	Small	**−**0.40	**−**0.11
	Rmssd30	**−0.66**	Large	**−**0.74	**−**0.57	0.07	Trivial	**−**0.08	0.22	**−0.28**	Small	**−**0.41	**−**0.13	**−0.34**	Moderate	**−**0.47	**−**0.19
	Rmssd60	**−0.72**	Very large	**−**0.79	**−**0.64	0.12	Small	**−**0.04	0.27	**−0.39**	Moderate	**−**0.51	**−**0.25	**−0.46**	Moderate	**−**0.58	**−**0.34
Exercise	Rmssd	**−0.51**	Large	**−**0.62	**−**0.39	0.13	Small	**−**0.02	0.28	**−**0.10	Trivial	**−**0.25	0.06	**−0.21**	Small	**−**0.35	**−**0.05
	RPE	**0.66**	Large	0.57	0.74	0.02	Trivial	**−**0.13	0.17	**0.50**	Large	0.37	0.60				
	Rf	**0.54**	Large	0.42	0.64	0.00	Trivial	**−**0.16	0.15								
	SpO_2_	**−0.10**	Small	**−**0.24	0.06												

HR, Heart rate; HRR, Heart rate recovery; nHRR, normalized heart rate recovery; RMSSD, Root mean square of successive differences of R–R intervals; SpO_2_, pulse oxygen saturation; Rf, respiratory frequency, RPE, Rate of perceived exertion

Bold characters denote significant correlations (*p* < 0.05)

Similarly, RMSSD30 and RMSSD60 exhibited large and very large correlations, respectively, with HRbout. Additionally, nHRR indices and RMSSD during recovery were negatively correlated with both Rf and RPE, showing small to moderate strengths (all *p* < 0.05). In contrast, there were positive correlations, albeit of lower magnitude (small), between cardiac nHRR indices and SpO2. It is noteworthy that SpO2 did not show significant correlations with HRV recovery indices (all *p* > 0.05).

### Post-exercise responses

The comprehensive cardiac autonomic and cardiovascular post-exercise responses can be found in Tables 2 and 3, respectively.

**Table 2:** Cardiac autonomic responses

		FiO_2_ = 16.2% (≈2000 m)			FiO_2_ = 14.3% (≈3000 m)			FiO_2_ = 12.6% (≈4000 m)		
		PRE	POST**−**15 min	POST-60 min	PRE	POST-15 min	POST-60 min	PRE	POST-15 min	POST-60 min
meanRR	(ms)	1098 ± 165	964 ± 118^*^	1087 ± 174	1097 ± 220	993 ± 154^*^	1112 ± 211	1120 ± 196	1023 ± 158^*^	1078 ± 152
Ln-SDNN	(ms)	4.30 ± 0.49	4.35 ± 0.41	4.49 ± 0.49^$^	4.35 ± 0.36	4.51 ± 0.52	4.41 ± 0.55^$^	4.33 ± 0.43	4.41 ± 0.39	4.54 ± 0.45^$^
Ln-RMSSD	(ms)	4.23 ± 0.54	4.08 ± 0.57	4.32 ± 0.52^$^	4.20 ± 0.46	4.21 ± 0.62	4.26 ± 0.63^$^	4.24 ± 0.58	4.13 ± 0.55	4.29 ± 0.46^$^
pNN50	(%)	44 ± 22	37 ± 20	47 ± 21	44 ± 20	40 ± 24	44 ± 23	43 ± 23	39 ± 21	46 ± 19
HF_peak_	(Hz)	0.23 ± 0.06	0.21± 0.06	0.22 ± 0.06	0.22 ± 0.06	0.21 ± 0.05	0.23 ± 0.06	0.25 ± 0.06	0.22 ± 0.07	0.21 ± 0.05
Ln-HF	(ms^2^)	7.25 ± 0.97	7.04 ± 1.13	7.50 ± 0.96	7.27 ± 0.88	7.48 ± 1.19	7.41 ± 1.20	7.19 ± 1.04	7.12 ± 1.08	7.39 ± 0.80
Ln-LF	(ms^2^)	7.43 ± 0.94	7.50 ± 1.06	7.82 ± 1.07	7.37 ± 0.59	7.77 ± 0.86	7.52 ± 1.16	7.35 ± 1.28	7.65 ± 0.76	7.80 ± 0.96
Ln-VLF	(ms^2^)	7.24 ± 1.19	7.42 ± 1.03	7.48 ± 1.29	7.13 ± 0.97	7.70 ± 1.52	7.64 ± 1.29	7.49 ± 0.89	7.69 ± 1.01	7.89 ± 1.13
HF n.u	(%)	46 ± 11	39 ± 11	43 ± 12	48 ± 13	43 ± 15	47 ± 19	46 ± 14	38 ± 13	41 ± 16
LF n.u	(%)	54 ± 11	61 ± 11	57 ± 12	52 ± 13	57 ± 15	53 ± 19	54 ± 14	62 ± 13	59 ± 16
Ln-LF/HF	(-)	0.82 ± 0.27	0.97 ± 0.27	0.90 ± 0.32	0.78 ± 0.35	0.90 ± 0.40	0.83 ± 0.42	0.82 ± 0.28	1.03 ± 0.36	0.97 ± 0.39
TP	(ms^2^)	8.49 ± 0.96	8.51 ± 0.98	8.80 ± 0.99	8.45 ± 0.71	8.94 ± 1.08	8.71 ± 1.14	8.52 ± 0.98	8.67 ± 0.85	8.94 ± 0.88

RR, R-R intervals; SDNN, standard deviation of R-R intervals; RMSSD, Root mean square of successive differences of R–R intervals; pNN50: Ln, natural logarithm transformation; pnn50, percentage of successive normal interbeat intervals greater than 50 ms; HF, High-frequency spectral power; LF, Low-frequency spectral power; TP, Total spectral power; n.u. normalized unit

^*^ = significantly different from PRE general time effect

^$^ = significantly different from POST-15 min general time effect; [two-way (time × group) repeated measures ANOVA followed by Holm–Sidak post hoc analyses; *p* < 0.05

**Table 3:** Cardiovascular responses

		FiO_2_ = 16.2% (≈2000 m)			FiO_2_ = 14.3% (≈3000 m)			FiO_2_ = 12.6% (≈4000 m)		
		PRE	POST-15 min	POST-60 min	PRE	POST-15 min	POST-60 min	PRE	POST-15 min	POST-60 min
*Hemodynamics*										
SBP	mmHg	117 ± 11	107 ± 9^*^	121 ± 12	114 ± 12	104 ± 8^*^	114 ± 13	114 ± 8	105 ± 8^*^	116 ± 11
DBP	mmHg	51 ± 13	49 ± 8	55 ± 12$	50 ± 11	45 ± 9	53 ± 9$	48 ± 6	48 ± 7	55 ± 10$
MAP	mmHg	73 ± 11	68 ± 8^*^	77 ± 11$	71 ± 10	65 ± 7^*^	74 ± 9$	70 ± 4	67 ± 4^*^	75 ± 9$
HR	bpm	56 ± 8	63 ± 8^*^	57 ± 9	57 ± 11	62 ± 10^*^	56 ± 10	56 ± 9	60 ± 8^*^	57 ± 75
CO	L*min^−1^	5.4 ± 1.3	5.4 ± 1.0	5.6 ± 1.7	5.6 ± 1.2	5.3 ± 1.0	5.4 ± 1.4	5.3 ± 1.0	4.9 ± 0.9	5.2 ± 0.9
SV	mL	97 ± 26	85 ± 14^*^	98 ± 24	99 ± 25	86 ± 15^*^	96 ± 24	95 ± 12	82 ± 10^*^	91 ± 11
TPR	mmHg·min*L^−1^	14.2 ± 3.9	13.1 ± 2.7	14.7 ± 3.9	13.5 ± 4.2	12.4 ± 2.3	14.4 ± 3.7	13.7 ± 2.7	14.0 ± 2.4	14.8 ± 2.6
*Spontaneous Baroreflex Sensitivity*										
Nseq-		11 ± 4	14 ± 3	11 ± 5	11 ± 3	14 ± 5	12 ± 3	12 ± 4	12 ± 4	12 ± 6
Nseq +		12 ± 4	12 ± 4	10 ± 5	11 ± 3	14 ± 4	11 ± 3	10 ± 3	11 ± 4	13 ± 7
Ln-BRSseq-	ms*mmHg^−1^	2.46 ± 0.64	2.63 ± 0.42	2.88 ± 0.57	2.64 ± 0.46	2.77 ± 0.51	2.88 ± 0.52	2.62 ± 0.43	2.62 ± 0.54	2.58 ± 0.55
Ln-BRSseq +	ms*mmHg^−1^	2.52 ± 0.54	2.69 ± 0.52	2.86 ± 0.49	2.58 ± 0.34	2.89 ± 0.44	2.86 ± 0.55	2.58 ± 0.60	2.64 ± 0.66	2.65 ± 0.43
Ln-BRSseq_MEAN_	ms*mmHg^−1^	2.49 ± 0.57	2.66 ± 0.46	2.88 ± 0.50	2.64 ± 0.37	2.83 ± 0.46	2.89 ± 0.48	2.62 ± 0.48	2.64 ± 0.57	2.64 ± 0.46

SBP, systolic blood pressure; DBP, diastolic blood pressure; MAP, mean arterial pressure; HR, heart rate; CO, cardiac output; SV, stroke volume; TPR, total peripheral resistance, BRS, Baroreflex sensitivity; seq + , positive sequences; seq-, negative sequences. N, number of sequences

^*^ = significantly different from PRE general time effect

^$^ = significantly different from POST-15 min general time effect; [two-way (time × group) repeated measures ANOVA followed by Holm–Sidak post hoc analyses; *p* < 0.05

Statistical analyses revealed a significant time effect (*p* < 0.001) on meanRR, with no significant influence from condition (*p* = 0.665) or interaction (*p* = 0.404) effects. MeanRR decreased at POST-15 compared to PRE across all conditions, indicating a general time effect (*p* < 0.05).

Additionally, a significant time effect (*p* < 0.05) was observed for SDNN and RMSSD, both of which increased at POST-60 compared to POST-15 (*p* < 0.05). However, no significant effects of exercise protocols were noted on other HRV indices.

A significant time effect (*p* < 0.001) was observed on SBP, with no significant influence from condition (*p* = 0.143) or interaction (*p* = 0.935) effects. SBP decreased at POST-15 compared to PRE consistently across all three exercise sessions, indicating a general time effect (*p* < 0.05).

Similarly, a significant time effect (*p* = 0.008) was noted on DBP, without significant condition (*p* = 0.653) or interaction (*p* = 0.619) effects. DBP increased at POST-60 compared to POST-15 across all conditions, reflecting a general time effect (*p* < 0.05).

Moreover, a significant time effect (*p* = 0.004) was observed on MAP, with no significant condition (*p* = 0.468) or interaction (*p* = 0.882) effects. MAP decreased at POST-15 and increased at POST-60 compared to PRE, suggesting a general time effect (*p* < 0.05).

Regarding SV, a significant time effect (*p* < 0.001) was reported, while no significant condition (*p* = 0.420) or interaction (*p* = 0.894) effects were observed. SV decreased at POST-15 compared to PRE, indicating a time effect (*p* < 0.05).

**Fig. 3:**
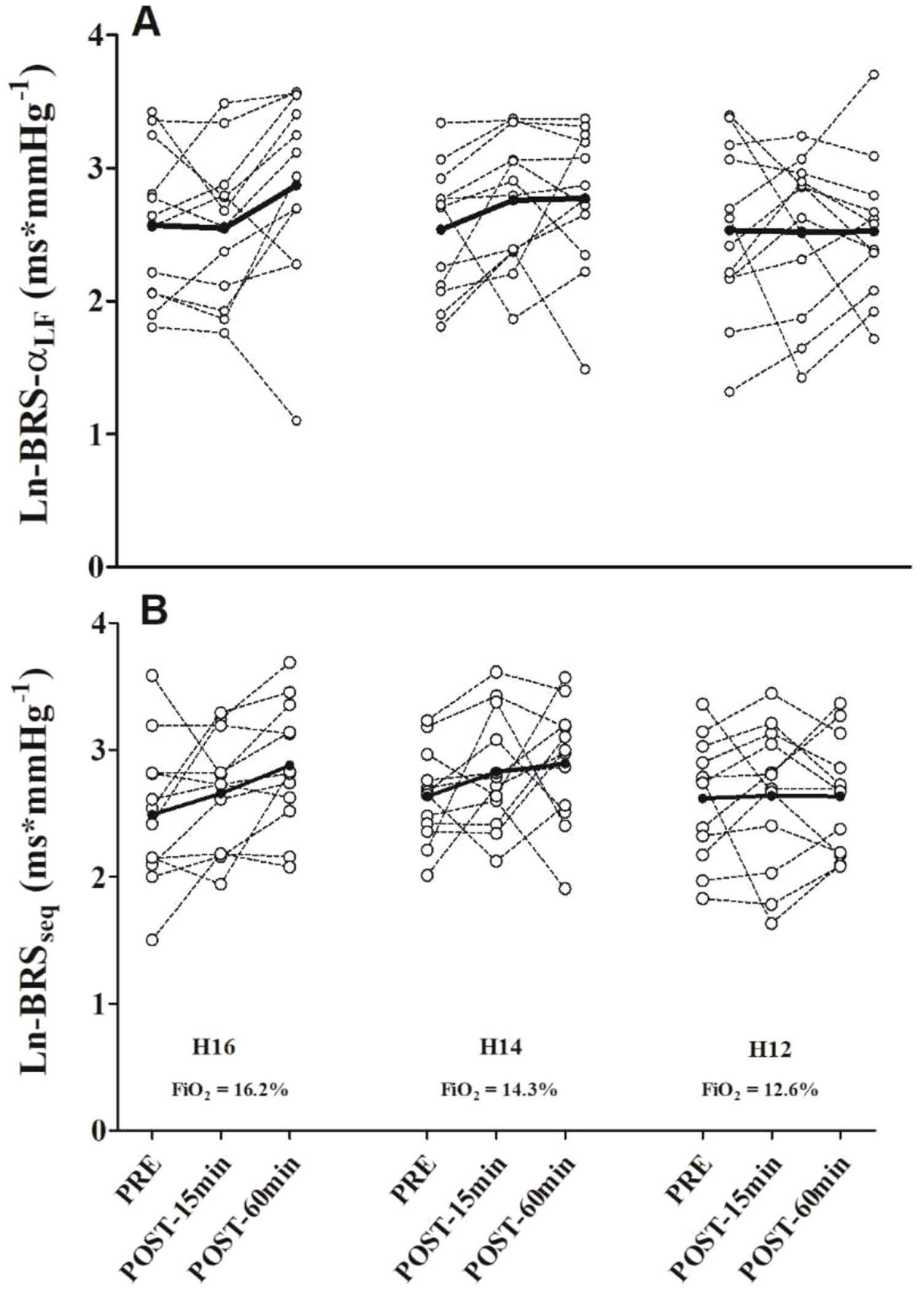
Individual Baroreflex sensitivity responses to the three investigated exercise protocols. Panel A: spectral method for baroreflex sensitivity assessment; Panel B: sequence method

However, no significant effects of time (*p* = 0.580), condition (*p* = 0.277), or interaction (*p* = 0.746) were noted on CO. Likewise, there were no significant effects of time (*p* = 0.101), condition (*p* = 0.352), or interaction (*p* = 0.532) on BRS parameters (Fig. 3).

## Discussion

The aim of this study was to examine the effect of performing HR-matched exercise with different degrees of hypoxic stress on the exercise and post-exercise autonomic and cardiovascular responses. The key findings of this study are that: 1) despite the progressive decrease in oxygen saturation (SpO_2_) with increasing levels of hypoxia (from 16.2% to 12.6% of FiO_2_), interval-type HR-matched exercise (75%HRmax) results in similar ventilatory (i.e., respiratory frequency), perceptual (i.e., RPE) and within-session cardiac autonomic responses (i.e., HR and HRV recovery) regardless of the degree of hypoxic stress; 2) the comparable exercise responses translate into similar post-exercise autonomic and cardiovascular responses. Baseline cardiac autonomic activity and hemodynamics were completely recovered within 60 min after HE cessation in all the three investigated conditions (FiO2 = 16.2%, 14.3%, 12.6%), while BRS was not affected at all.

### Exercise responses

Previous studies have demonstrated a clear association between disturbances in homeostasis induced by exercise and the subsequent recovery of cardiac autonomic function. Exercise-related parameters such as elevated exercise intensities and longer durations, along with external factors like challenging environmental conditions, have been associated with heightened perturbation of homeostasis induced by exercise. This often leads to delayed recovery of HRR and HRV. Both indices are valid tools to investigate the disturbance induced in ANS balance by an acute bout of exercise. Particularly, vagal-related HRV indices, such as RMSSD and HF, are the most employed indices to investigate post-exercise autonomic recovery (Buchheit et al. 2007; Peçanha et al. 2017; Michael et al. 2017).

The balance between sympathetic and parasympathetic activity shifts as the exercise session progresses and during short-periods of recovery throughout the interval exercise. Changes in HRR and HRV at different stages of an interval exercise session provide valuable insight into the ability of the cardiovascular system to cope with exercise stress and its recovery efficiency, which may vary depending on the type of exercise and the characteristics of the subjects (Seiler et al. 2007a, 2007b). As the session progresses, the cardiovascular system dynamically adapts to the varying levels of sympathetic nervous activity. This adaptability is evident in the HRV measurements (RMSSD30 and RMSSD60) following the second bout, preceding the HRR changes (HRR30 and HRR60) after the third bout. Differences in timing between HRV decrease and HRR change suggest a complex interplay between the immediate stress response and subsequent recovery mechanisms. HRV changes reflect the immediate response to exercise stress involving a shift towards decreased parasympathetic activity and increased sympathetic activity. As a result, the cardiovascular system is under increased strain, and the recovery mechanisms from previous bouts are less effective due to cumulative fatigue. Insufficient recovery can lead to an increased dominance of sympathetic activity and/or a larger and/or sustained decrease in parasympathetic activity as the session progresses, which is associated with the progressive rise in heart rate observed during exercise.

On the other hand, hypoxic conditions may exacerbate the sympathetic response, leading to an increase in HR, as they impose additional stress on the cardiovascular system during exercise. At altitudes above 3500 m, hypoxia may impair autonomic nervous system (ANS) function due to the significant respiratory effort required leading to a hyperventilation response and exacerbating sympathetic activity (Al Haddad et al. 2012). However, according to the results of this study, HR and HRV remained unaffected by variations in hypoxic conditions. Despite decreasing SpO_2_ levels with increasing degrees of hypoxic stress (FiO_2_ = 16.2% ≈2000 m a.s.l. (H16); FiO_2_ = 14.3% ≈3000 m a.s.l. (H14); FiO2 = 12,6% ≈4000 m a.s.l. (H12)), subjects exhibited comparable respiratory frequencies, RPE and cardiac autonomic responses across all conditions, suggesting comparable ventilatory effort. However, as minute ventilation, respiratory frequency and tidal volume were not directly assessed, this interpretation should be taken with caution.

In light of all these findings, hypoxia-induced increase in cardiovascular and ventilatory stimulation has been shown to be controllable by matching heart rate responses (Fornasiero et al. 2021, 2019; Chacaroun et al. 2019). According to Chacaroun et al. (Chacaroun et al. 2019), the primary difference in cardiac autonomic function between normoxia and hypoxic heart rate-matched exercise is the elevated HR observed during recovery phases of interval exercise in hypoxia. This suggests a slower recovery rate under hypoxic conditions, as mentioned earlier. Indeed is the strong relationship observed between the progressive increase in RPE and Rf and the decrease in cardiac autonomic recovery indices (e.g., HRR and RMSSD) during hypoxic interval exercise suggest an increase of fatigue. Therefore, specific protocol adjustments should be considered when training is performed under hypoxic conditions, regardless of level of hypoxia. For example, progressively longer short recovery bouts during hypoxic interval training may be necessary to allow heart rate stabilization, which would support more effective recovery and enhance performance outcomes.

### Post-exercise responses

In the post-exercise period, HR, HRV and BP responses reveal the impact of previous exercise stimulus on autonomic disturbance and subsequent recovery. During exercise, HR increases and HRV decreases, with different contributions from parasympathetic withdrawal and sympathetic activation. After exercise, HR progressively decreases and HRV indices tend to return to baseline levels (i.e., post-exercise parasympathetic reactivation and sympathetic withdrawal) (Peçanha et al. 2017). The speed of cardiac autonomic recovery after exercise cessation is directly influenced by the intensity of the exercise (Michael et al. 2017). In general, moderate exercise intensities (i.e., below the first ventilatory threshold, VT) are associated with a fast recovery of cardiac autonomic indices in the post-exercise period, however, exercise sessions above VT require more time for the recovery to occur.

In our study, with moderate intensity exercise, we observed a recovery of vagal-related indices at 60 min post-exercise cessation. This suggests a fast recovery of cardiac autonomic function during the post-exercise phase. The decrease in mean RR at POST-15 compared to PRE, may be partially influenced by the temporary elevation in core temperature associated with the exercise session and may do not directly attest autonomic imbalance. The increased heart rate, manifested by a shorter RR interval, is part of the body’s thermoregulatory response by increasing the volume of blood circulating through the body each minute.

The increase in SDNN and RMSSD at 60 min post-exercise indicates parasympathetic recovery, reflected in a gradual and controlled modulation of heart rate without abrupt changes in autonomic activity, as demonstrated by the stability of pNN50. The absence of changes in frequency domain indices (HF, LF, LF/HF and VLF) suggests that recovery is occurring more at the level of short-term modulation of heart rate (SDNN, RMSSD), without indicating a change in the overall autonomic balance, as it is only a single exercise session (Task Force of the European Society of Cardiology et al. 1996).

On the other hand, the autonomic nervous system's control over heart function during recovery remains stable between different hypoxic levels. Despite the additional stress that hypoxia condition may add to the exercise, heart may adapt to hypoxic stress without significant disruption to its function during recovery, at least within the specific conditions tested (HR-matched exercise). Indeed, in a previous study using the same protocol, we observed a similar autonomic response to a normoxic protocol, which supports our hypothesis (Fornasiero et al. 2021). Indeed HRV response may be impair in hypoxic conditions depending on the intensity of exercise (Al Haddad et al. 2012). By using heart rate-adapted exercises, cardiovascular effort is kept constant, even when oxygen availability (FiO_2_) varies. As a result, hypoxia does not seem to significantly affect recovery dynamics. Adaptation and efficiency of vagal reactivation (parasympathetic response) appear comparable after similar workloads (post-exercise response).

Concurrently, the decrease in SV at POST-15 compared to PRE may reflect a transient cardiovascular adjustment after exercise cessation. However, without direct measurements of left ventricular volumes, we cannot conclusively determine the underlying mechanism, such as changes in preload or contractility. The observed changes in SV and meanRR interval contribute to maintaining a consistent CO response during post-exercise recovery. Initially, the decrease in SV may be compensated for by an elevated heart rate, inferred from the shortening of mean RR interval, leading to a balanced hemodynamic response. Subsequently, the restoration of SV and HR to pre-exercise levels by 60 min suggests a completely reestablished hemodynamics.

Changes in blood pressure after exercise are consistent or in agreement with the SV dynamics discussed above. The initial decrease in blood pressure values at POST-15 reflects the so-called post-exercise hypotension (PEH), indicative of an immediate PEH response due to factors such as reduced sympathetic activity and vasodilation and transient reduction in vascular resistance (Halliwill et al. 2013). The subsequent increase in MAP at POST-60 suggests the restoration of vascular tone as the body progresses further from the immediate post-exercise period, which is coherent with the observed increase in DBP at POST-60 compared to POST-15 during the recovery period (Kaufman et al. 1987).

Post-exercise BRS seems to be dependent upon previous exercise intensity (Reynolds et al. 2017). Previous research has demonstrated enhancements in BRS following different durations and intensities of exercise. For instance, BRS has been shown to improve after a 30-min session at 65% of HRmax and following 60 min of moderate exercise (approximately 75% of HRmax) (Raczakv et al. 2005; Terziotti et al. 2001). On the contrary, high-intensity (> 85% HRmax) and maximal exercise have been found to reduce BRS, with recovery typically occurring within 60 min following exercise cessation (Halliwill et al. 2013).

In this study, no significant effects were observed on CO or BRS parameters, indicating a stable response during the post-exercise recovery phase, despite the physiological stress of exercise, helping to maintain cardiovascular homeostasis. This suggests that the cardiovascular system can adapt effectively to low-oxygen conditions if the exercise is tailored to maintain heart rate levels similar to those observed in normoxic conditions (Fornasiero et al. 2021, 2019), but for shorter durations (Fornasiero et al. 2021). This adaptation is crucial for maintaining performance levels and preventing premature fatigue or performance decline.

Several limitations should be considered. Our findings are specific to the exercise protocol used in this study. Variations in exercise type, intensity, and duration, along with more severe hypoxic conditions, could yield diverse autonomic and cardiovascular responses (Halliwill et al. 1996). Additionally, the posture adopted during recovery (e.g., sitting versus supine) may influence cardiovascular and autonomic responses. Consequently, the findings of this study may be confined to the specific exercise protocol and recovery methods utilized by the participants. Furthermore, the lack of direct assessments of muscle sympathetic nerve activity (MSNA) or blood catecholamine responses limits our ability to fully assess sympathetic influence on the observed outcomes. However, previous studies have well characterized sympathetic activation during dynamic exercise (Ichinose et al. 2008; Katayama & Saito 2019). These findings suggest that a sympathetically mediated response may have contributed to the cardiovascular adjustments observed during and after exercise. Similarly, we did not measure exercise-induced metabolite accumulation, such as blood lactate concentration, which provides insights into metaboreflex and sympathetic activation levels during exercise. Lastly, the study participants consisted of healthy, active young men, potentially limiting the generalizability of these findings to sedentary, clinical, or elderly populations.

## Conclusions

Despite decreasing oxygen saturation with increasing hypoxic stress levels, variations in hypoxia levels ranging from 2000 to 4000 m a.s.l. do not impact the outcomes concerning ventilatory, perceptual, and cardiac autonomic responses during both exercise and post-exercise recovery phases. This finding has several important implications. On one hand, the results of this study highlight the adaptability of the cardiovascular system to heart rate-matched exercise under varying degrees of hypoxic stress, which minimize the risk of adverse events. On the other hand, the stable ventilatory and cardiac autonomic responses observed between 2000 and 4000 m a.s.l. during interval-type HR-matched hypoxic exercise offer significant flexibility for the development of personalized training programs and the implementation of recovery strategies to prevent overload, injuries, and optimize performance.

## Data Availability

The data that support the findings of this study are available from the corresponding author upon reasonable request.

## Abbreviations

ANOVA: Analysis of variance
BRS: Baroreflex sensitivity
BRSSeq+: Positive sequence of baroreflex analysis
BRSSeq-: Negative sequences of baroreflex analysis
DBP: Diastolic blood pressure
Fast: Fourier Transform
FiO_2_: Fraction of inspired oxygen
H12: Hypoxia level at FiO2 = 12.6% (≈4000 m a.s.l; H12)
H14: Hypoxia level at FiO2 = 14.3% (≈3000 m a.s.l; H14)
H16: Hypoxia level at FiO2 = 16.2% (≈2000 m a.s.l; H16)
HF: High frequency (HF, 0.15–0.4 Hz)
HE: Hypoxic exercise
HR: Heart rate
HR: Max Maximal heart rate
HR30: Heart rate mean of the last 30 s of recovery phase between bouts
HR60: Heart rate mean of the last 60 s of recovery phase between bouts
HRbout: Heart rate at the end of exercise
HRR: Post-exercise heart rate recovery
HRV: Heart rate variability
IBI: Inter-beat interval
LF: Low frequency (LF, 0.04–0.15 Hz)
NE: Normoxic exercise
nHRR: Normalized heart rate recovery
nHRR30: Normalized heart rate recovery of the last 30 s of recovery phase between bouts
nHRR60: Normalized heart rate recovery of the last 60 s of recovery phase between bouts
PEH: Post-exercise hypotension
pNN50: Percentage of successive R-R intervals differing more than 50 ms from the previous interval
POST-15 min: Recovery-15 min post-exercise
POST-60 min: Recovery-60 min post-exercise
PRE: Before exercise
Rf: Respiratory frequency
RMSSD: Root mean square of successive differences of R-R intervals
RMSSD30: Root mean square of successive differences of R-R intervals (RMSSD) was calculated for the last 30 s of exercise
RMSSD60: Root mean square of successive differences of R-R intervals (RMSSD) was calculated for the last 60 s of exercise
RPE: Rating of perceived exertion
SBP: Systolic blood pressure
SDNN: Standard deviation of normal-to-normal RR intervals
SpO2: Oxygen saturation
TP: Total power (TP, 0–0.4 Hz)
